# Polymer Composites Based on Glycol-Modified Poly(Ethylene Terephthalate) Applied to Additive Manufacturing Using Melted and Extruded Manufacturing Technology

**DOI:** 10.3390/polym14081605

**Published:** 2022-04-14

**Authors:** Katarzyna Bulanda, Mariusz Oleksy, Rafał Oliwa

**Affiliations:** Department of Polymer Composites, Faculty of Chemistry, Rzeszów University of Technology, Al. Powstańców Warszawy 6, 35-959 Rzeszów, Poland; oliwa@prz.edu.pl

**Keywords:** PET-G, composites, hybrid materials, blends, additive manufacturing, MEM, 3D printing

## Abstract

As part of the work, innovative polymer composites dedicated to 3D printing applications were developed. For this purpose, the influence of modified fillers, such as silica modified with alumina, bentonite modified with quaternary ammonium salt, and hybrid filler lignin/silicon dioxide, on the functional properties of composites based on glycol-modified poly(ethylene terephthalate) (PET-G) was investigated. In the first part of the work, using the proprietary technological line, filaments from unfilled polymer and its composites were obtained, which contained modified fillers in an amount from 1.5% to 3.0% by weight. The fittings for the testing of functional properties were obtained using the 3D printing technique in the Melted and Extruded Manufacturing (MEM) technology and the injection molding technique. In a later part of the work, rheological properties such as mass melt flow rate (MFR) and viscosity, and mechanical properties such as Rockwell hardness, Charpy impact strength, and static tensile strength with Young’s modulus were presented. The structure of the obtained composites was also described and determined using scanning electron microscopy with an attachment for the microanalysis of chemical composition (SEM/EDS) and the atomic force microscope (AFM). The correct dispersion of the fillers in the polymer matrix was confirmed by wide-angle X-ray scattering analysis (WAXS). In turn, the physicochemical properties were presented on the basis of the research results: thermogravimetric analysis (TGA), differential scanning calorimetry (DSC), and Fourier transform infrared spectroscopy (FT-IR). On the basis of the obtained results, it was found that both the amount and the type of fillers used significantly affected the functional properties of the tested composites.

## 1. Introduction

Additive manufacturing (AM), also known as three-dimensional printing (3DP), is a groundbreaking, still innovative technology that enables the realization of original goals in terms of material development, design, and production techniques. AM has a positive effect on the industry and economy, as it allows the reduction in the amount of post-production waste, increasing the efficiency of processes and thus reducing costs [1,2,3]. Additive manufacturing was initially limited to small-part prototyping, but now, the impressively rapid advancement of technology has allowed for the development of potential AM engineering applications in industry. Scientists all over the world are working on the development of geometrically complex high-quality elements from various materials, including innovative biomaterials [1,2], while maintaining high precision and printing efficiency [3,4,5].

AM can be grouped into seven main categories, depending on the different printing principles [6,7]: powder bed fusion, binder jetting, sheet lamination, direct energy deposition, material jetting, vat photo-polymerization, and material extrusion. Material extrusion (ME) is an additive manufacturing technique in which the filament material is pressed through the print head by mechanical force and then selectively deposited to build a predetermined model. ME technology is one of the older 3D printing techniques. It was originally developed for design applications and the creation of functional prototypes but has now gained considerable recognition in the industry for its affordability, process simplicity, and the ability to manufacture parts from a range of commonly used thermoplastics [8,9]. One of the ME techniques is Melted and Extruded Manufacturing (MEM) technology, which consists in applying a layer of material heated to about 0.5 °C above the melting point, which is then extruded through a die of a specific diameter so that it solidifies about 0.1 s after extrusion by welding with the previous layers [10,11]. The material used is in the form of a wire of a specific diameter (filament), which allows the material to be continuously dosed into the melting chamber [12]. The printer head consists of a motor, a melting coil, and the aforementioned nozzle. While obtaining a single layer, the head moves in the XY plane over the part. Another layer can be applied by lowering the build plate or by lifting the nozzle. The entire process is computer-controlled through specialized software [13].

Materials for 3D printing are most often selected due to the properties of the raw material, the planned final use of the model obtained, but also due to the type of available equipment, because MEM printers do not process all types of available materials [14]. Still, the biggest problem is the mechanical properties of the obtained products, which do not match the details made with standard methods, such as injection-molded, because the 3D elements are less homogeneous, they are characterized by empty spaces between the printed threads [15]. Designers most often mention the cost of the process, as well as the cost of the raw material, as another criterion for selecting a material for MEM processes. The material intended for 3D printers should have several characteristic features: safety, nontoxicity, compatibility, sufficient viscosity of the material after melting (this is the only way to bond the material layer by layer), low glass transition temperature of the material, and low melting point, which results in a lower consumption of energy during the process [16,17]. MEM printers can produce parts from materials and thermoplastics such as PLA (polylactide), ABS (acrylonitrile butadiene styrene), PC (polycarbonate), and PC/ABS-mixed materials [12]. Additive production was initially focused solely on polymers, which were then replaced or supplemented by composites, ceramics, and metals. The use of polymer composites in AM technology allows the obtaining of the desired performance properties of the product and often facilitates the production process.

Poly(ethylene terephthalate) also turned out to be an interesting material for 3D printing. Currently, a lot of emphasis has been placed on the correction of the properties of PET fibers, which is why filaments are most often made of a glycol-modified version of the polymer (PET-G) [18]. Modification of the material with glycol allows fibers to be obtained that are less brittle, easier to print, and more transparent in the case of transparent versions of this material. PET-G forms a durable material with good mechanical properties [19]. The material has a low thermal expansion; therefore, it is an ideal raw material for the production of complex details and, above all, larger models. PET-G can be used for both external and internal applications, and in addition, the material is suitable for the production of industrial and mechanical tools and parts. So far, scientists have already studied the influence of the most important printing parameters on the performance of products obtained from PET-G. Kam and Ipekci in their work [20] with the Taguchi method determined the influence of the layer thickness and the raster angle on the mechanical behavior of the prepared details. The results showed that the optimal 3D printing parameters in terms of PET-G material strength were the layer thickness of 0.25 mm and the screen angle of 45 degrees. In contrast, Basurto-Vazquez et al. [21] investigated the effect of fill density (30%, 70%, and 100%) and printing orientation (edge, flat, and vertical) on the mechanical properties of the 3D-printed PET-G honeycomb structure. The structure with a vertical printing direction and 100% fill density showed the desired performance and was characterized by high energy absorption. In order to increase the adhesion of 3D-printed parts from glycol-modified poly(ethylene terephthalate), Silva et al. [22] proposed the modification of sodium-neutralized poly(ethylene-methacrylic acid) (EMAA) in various compositions by weight of PET-G/EMAA, 70/30, 50/50, and 30/70. The results showed that the interaction between PET-G and EMAA was conducive to the production of 3D-printed elements with increased layer adhesion, as well as plasticity and strength, compared to PET-G, and the 30/70 and 50/50 blends were characterized by the best printability in terms of adhesion between printed layers and satisfactory mechanical properties.

In summary, there are few publications in the literature on the modification of the properties of the PET-G polymer intended for applications in 3D printing. As part of the work, polymer composites based on glycol-modified poly(ethylene terephthalate) with the addition of selected modified nanofillers and fillers were developed. The fillers were selected in order to improve, above all, the printability of PET-G, while maintaining good mechanical and physicochemical properties of the polymer. The research presented in this paper is a continuation of another work [23].

## 2. Materials and Methods

### 2.1. Materials

Commercial material (NOCTUO 3D Filaments, Gliwice, Poland) was used as the polymer matrix (designated as PET-G). PET-G was filled with: silica (S) containing alumina (Aerosil MOX 170, Evonic Industries, Hanau, Germany), bentonite (B) (technical product “Specjal” Zębiec SA Zakłady Górniczo-Metalowe, Zębiec, Poland) modified with quaternary ammonium salt (BARQUAT^®^ DM80, Lonza, Switzerland) and lignin (L) (Sigma-Aldrich, Burlington, MA, USA)/silicon dioxide (Syloid 244, WR Grace & Co., Columbia, MD, USA) hybrid filler. Detailed information on the procedure of obtaining bentonite modified with quaternary ammonium salt and lignin/silicon dioxide nanoparticles has already been patented and described before [24,25]. Modified fillers were introduced into PET-G in order to improve the flowability of composites (which significantly affects the processing properties of materials) and mechanical properties. Hybrid systems of fillers were introduced into the polymer in order to investigate the synergy effect of their operation and the impact on the functional properties of the obtained composites. Chemically modified polyethylene grafted with maleic anhydride (Fusabond E926, DuPont, Wilmington, DE, USA) was used as a compatibilizer.

The compositions of the individual compositions are summarized in Table 1.

### 2.2. Preparation of the Composite and Sample

**Figure 1:**
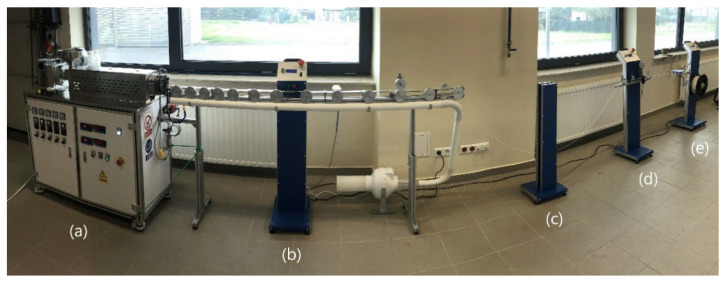
Proprietary technological line for filament production: (**a**) view of a single-screw extruder with a hopper, (**b**) view of an air-cooled roller extractor with a control panel, (**c**) filament extraction speed sensor system, (**d**) filament diameter measurement section, and (**e**) winder with control panel.

Before mixing the appropriate amount of the components of the individual compositions, the materials were dried in a vacuum oven (PET-G: 80 °C, 4 h; fillers S, B, L: 100 °C, 24 h). The ingredients of the composition were homogenized on a Coperion twin-screw extruder equipped with a granulating line with the following parameters: screw speed 400 rpm, extrusion capacity 4 kg/h, temperature from 180 °C to 220 °C. The granules thus obtained were dried in a vacuum oven at 80 °C for 4 h. The dried composites were used to obtain fibers with a diameter of approx. 1.75 ± 0.05 mm on the designed filament production line (Gamart SA, Jasło, Poland) in the extrusion temperature range from 190 °C to 220 °C (Figure 1).

**Figure 2:**
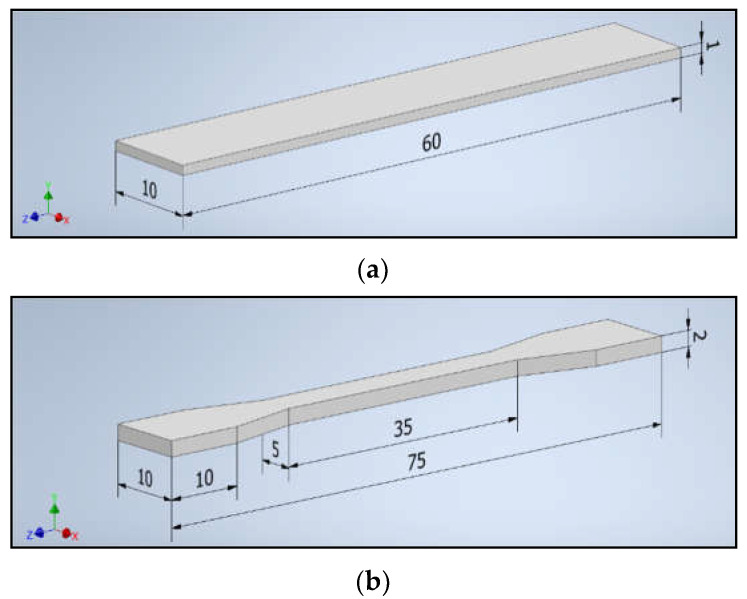
The dimensions of the samples: (**a**) a bar and (**b**) a paddle.

The composites were used to obtain samples (Figure 2) needed for further tests on the UP BOX (TierTime) 3D printer in the Melted and Extruded Manufacturing technology and by the injection method on the Haake MiniJet II mini-injection molding machine.

The process parameters are summarized in Table 2. 

### 2.3. Methods Characterization 

Melt flow rate, MFR was determined using a plastometer (DYNISCO 4781, Kayeness INC., Honey Brook, PA, USA). For this purpose, samples weighing about 4 g were introduced into the apparatus heated to 220 °C, and then a preload of 1.1 kg was applied for 240 s. After this time, the load was changed to the appropriate 2.16 kg and the measurements were started. The sample extruded from the nozzle was cut off after a predetermined time of 10 s, and then weighed. For each series, three measurements were carried out in accordance with the ISO 1133 standard.

Shear viscosity was measured using a capillary rheometer (Smart RHEO, Instron Ceast, Norwood, MA, USA). The samples weighing about 10 g were introduced into the suitably heated apparatus to 220 °C, where they were thermostated for 300 s under preload on the piston. The measurement was carried out for the shear rate ranges from 300 1s to 1000 1s. A capillary with a length of 40 mm and a width of 1.16 mm was used for the measurement. The test was performed in accordance with the standard 11443:2005.

Rockwell hardness was determined using a hardness tester (Zwick/Roell, Zwick GmbH & Co., Ulm, Germany) at ambient temperature. The sample was placed in the apparatus, a defined load was applied (load at which the indenter would collapse to a thickness of 0.15–0.35 mm), and a 30 s measurement was started. Ten determinations were carried out in accordance with the ISO 6508 standard for each series.

Charpy impact strength was determined using a hammer (PSW GEHARD ZORN, Stendal, Germany) with a force of 1 J. Samples for bar-shaped tests were prepared by notching the notch at 2 mm (CEAST-Instron, Via Airauda, Pianezza TO, Italy), and then placed horizontally on the supports of the machine in such a way that the hammer strikes the center of the edge of the specimen. For each series, five measurements were made in accordance with the ISP/179/1Ea standard.

The determination of the strength properties during the static tensile test was carried out on a testing machine (INSTRON 5967, Grove City, PA, USA) at ambient temperature. The paddle-shaped samples were placed in the machine grips, Young’s modulus was measured at a given tensile speed of 5 mm/min (until 1% tensile strain was obtained), and the speed was increased to 50 mm/min. Five measurements were made for each series in accordance with ISO 527.

Analysis of the microstructure by means of an atomic force microscope was performed with an apparatus (NanoScope VIII, Bruker, Warsaw, Poland) using an RTESPA scanning needle, with a resonance frequency of 300 kHz and a spring constant of 20–80 N/m. Images were captured at a scan rate of 0.5 kHz and a resolution of 256 lines and then analyzed using the NanoScope Analysis software.

To observe the microstructure, brittle fractures obtained by impact fracture of the sample after cooling it in liquid nitrogen were used. The observations were made using the scanning electron microscope (Hitachi TM3000, Red Star Vietnam Co., Lid., Hanoi, Vietnam) with an energy-dispersive spectroscopy, EDS, microanalysis device. Before the observations, samples of brittle fractures of polymers and composites were sputtered with a layer of gold with palladium. The observations were made using a voltage of 5 keV.

Thermogravimetric analysis was performed with a TGA/DSC 1 (Mettler Toledo DSC 1 Star^e^ System, METTLER Toledo, Schwerzenbach, Switzerland) under a nitrogen atmosphere. For the first apparatus, samples weighing 5 mg were heated on platinum plates from 25 °C to 600 °C, at a rate of 10 °C/min. The results were analyzed using STAR^e^ software.

To determine the physicochemical properties of the tested polymer composites, tests were carried out using differential scanning calorimetry (Mettler Toledo DSC 1 Star^e^ System, METTLER Toledo, Schwerzenbach, Switzerland). Measurements were made in a helium atmosphere in airtight aluminum crucibles. About 6 mg of samples was heated from −90 °C to 300 °C at 10 °C/min, then cooled to −90 °C at 10 °C/min and reheated to 300 °C at 10 °C/min.

Measurements using wide-angle X-ray diffraction (WAXS) were performed using a diffractometer (NanoStar-U, Bruker Inc., Billerica, MA, USA) with a two-dimensional detector in transmission geometry. X-rays with a wavelength of 1.54 Å were produced by irradiating a copper lamp supplied with a voltage of 600 µA at 50 kV. Measurements were made at room temperature (about 22 °C). The scattering angle range was from 0° to 28°. Fillers and polymer composites with their addition were tested.

The chemical structure of the studied polymer materials was analyzed by Fourier transform infrared spectroscopy using a Nicolet 8700 spectrophotometer with a diamond ATR attachment. Each sample was scanned 128 times in the wavelength range of 4000–650 cm^−1^, and the absorption spectra were recorded. The results were analyzed using the OMNIC Spectra software.

## 3. Results and Discussion

MFR is an important rheological parameter characterizing the flow of polymers, because it significantly affects the properties of the molding process, both by injection and 3D printing [26]. The increase in the fluidity of materials is very beneficial in the case of better filling in the injection mold and denser printing in the case of 3D printing technology.

The average test results for the mass flow rate of composites based on glycol-modified poly(ethylene terephthalate) are summarized in the Table 3. On the basis of the obtained results of the flowability of the tested materials, it was found that the addition of modified fillers to PET-G caused an increase in the obtained MFR values. The highest result, by 45.63% higher compared to the unmodified matrix, was obtained for the composite with the addition of 3%S. The remaining materials were characterized by an increase in fluidity in the range from 21.25% to 40.23%. The literature confirms that the composites with the addition of mineral fillers show an upward trend in the value of the mass melt flow rate to 3% of the filler content [27,28].

**Figure 3:**
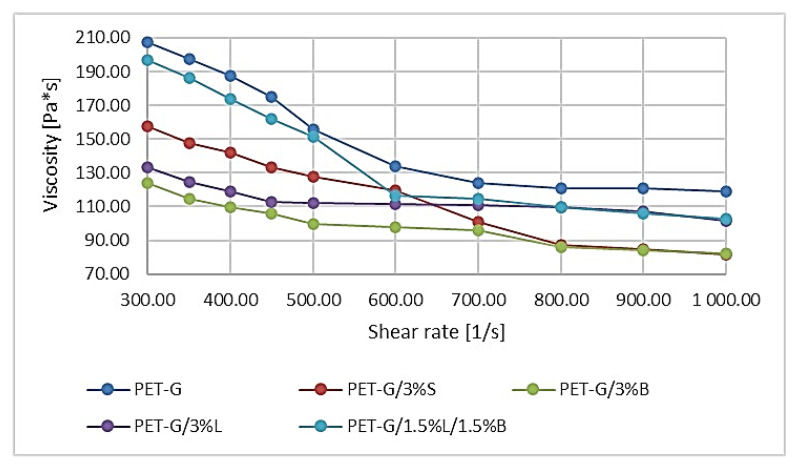
Viscosity curves of PET-G polymer and composites based on PET-G.

Knowledge of the rheological data of the tested materials is important in terms of proper design, as well as the proper conduct of processing processes. The available rheological properties are often limited only to the stated values of the mass melt flow rate (MFR), and less frequently to the viscosity curves. Based on the results of PET-G and its composites, it was found that the viscosity of the individual systems decreased with the increase in the shear rate. The introduction of modified fillers to the polymer matrix resulted in a decrease in the viscosity value of composites compared to unfilled poly(ethylene terephthalate). On the basis of the viscosity curves determined, the polymer with the highest viscosity, regardless of the shear rate, turned out to be PET-G, because the polymer is characterized by the lowest flowability. The addition of the hybrid system 1.5%L/1.5%B caused the viscosity to decrease by about 20 Pa ∗ s at 300 s^−1^ and at 1000 s^−1^ (Figure 3). On the other hand, the lowest viscosity was obtained for the composite filled with modified bentonite PET-G/3%B, which achieved 127.35 Pa ∗ s at 300 s^−1^ and 135.76 Pa ∗ s at 1000 s^−1^ (Figure 3).

When analyzing the results of research on the mechanical properties of composites obtained with the following methods: additive manufacturing and, for comparison, injection molding, it can be noticed that samples obtained by 3D printing generally had worse mechanical properties. For this reason, the results obtained with the two techniques are presented separately. The observation is widely described in the literature [29,30,31] and results from the better homogeneity of the samples obtained by injection molding [32].

**Figure 4:**
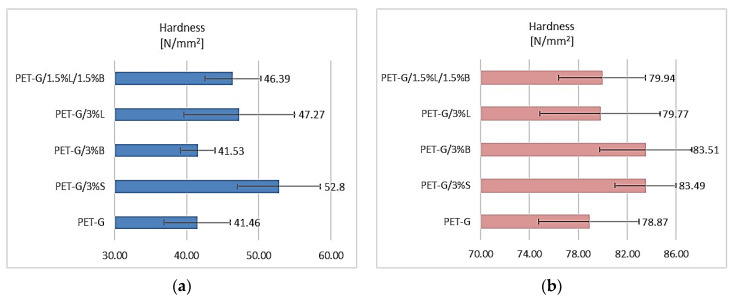
Hardness test results of (**a**) samples obtained by 3D printing and (**b**) samples obtained by injection molding.

It was noted that the introduced fillers had a positive effect on the obtained hardness results of fittings made using the rapid prototyping technology for PET-G and composites based on it (Figure 4a). The unmodified polymer had the lowest hardness, 41.46 N/mm^2^. The other polymer materials, apart from PET-G/3%B, achieved a hardness higher by 11.89–27.35% compared to PET-G. The best results were obtained for the PET-G/3%S composite, where the hardness was 52.80 N/mm^2^. The results of testing the hardness of fittings produced by injection molding are shown in Figure 4b. The introduced selected modified fillers allowed better test results to be obtained in comparison to the unmodified matrix. The best result was obtained for composites with the addition of modified silica (3% S; 83.49 N/mm^2^) and modified bentonite (3% B; 83.51 N/mm^2^).

**Figure 5:**
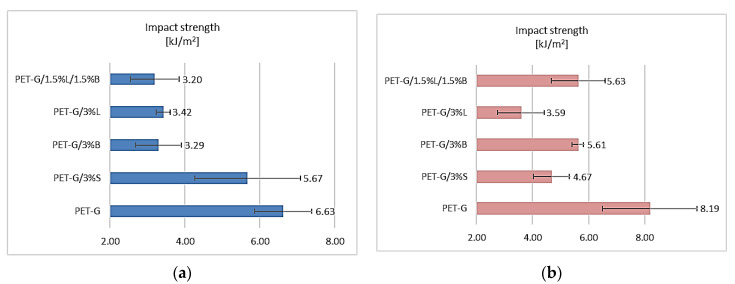
Impact strength test results of (**a**) samples obtained by 3D printing and (**b**) samples obtained by injection molding.

The addition of S, B, L, and L/B fillers had a significant impact on the test results obtained for both the fittings obtained in the rapid prototyping technology and the injection molding method (Figure 5). Unfortunately, the introduction of the selected fillers into the polymer matrix negatively influenced the obtained results of the determination. This negative phenomenon can most probably be explained by the increase in the stiffness of the structure of these composites, which is confirmed by the research on determining the hardness according to Rockwell (Figure 4). Observing the results obtained for the fittings made by the 3D printing method, it was found that the presence of the selected fillers significantly decreased the impact toughness of the tested composites (Figure 5a). The lowest results were obtained for the PET-G/1.5%L/1.5%B composite, for which the impact strength decreased by 51.73%. Unfortunately, in the case of samples obtained by injection molding, the impact strength of the composites was also lower, at the level from 3.59 kJ/m^2^ to 5.63 kJ/m^2^ compared to unfilled PET-G (Figure 5b).

In the case of composites obtained by 3D printing, after introducing fillers B and the hybrid L/B system, the Young’s modulus was improved. For composites containing 3%S and 3%L, Young’s modulus was slightly lower. On the other hand, when analyzing the results of Young’s modulus for moldings produced by injection molding presented in Table 4, a beneficial effect of the addition of these fillers on the mechanical properties of the obtained composites was noticed. In each case, the Young’s modulus for the obtained materials was higher compared to PET-G. The best result was determined for the composite filled with 3%B. As is known, the addition of modified layered aluminosilicates has a positive effect on the improvement of Young’s modulus. As it results from the data on the determination of tensile stress and strain at break presented in Table 4 for composite samples produced in 3D technologies, it was observed that the introduced additives had a significant effect on the change in mechanical properties of the tested composites. An increase in tensile stress of 9.8–12.53 MPa and a decrease in strain at break ranging from 15.04% to 21.02% in relation to the unfilled polymer matrix were obtained. Only in the case of the composite containing 3% silica (PET-G/3%S) did the breaking stress decrease (Table 4). For composite samples produced by injection molding, a decrease in tensile stress compared to PET-G and an increase in strain at break were obtained (Table 4).

Phase images and topographies on the surface of glycol-modified poly(ethylene terephthalate) and composites on its matrix were analyzed using the AFM microscope.

**Figure 6:**
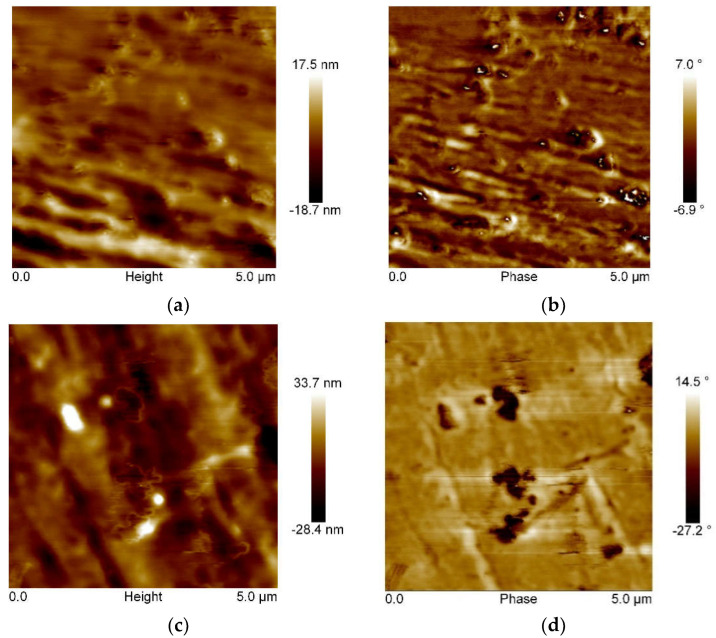

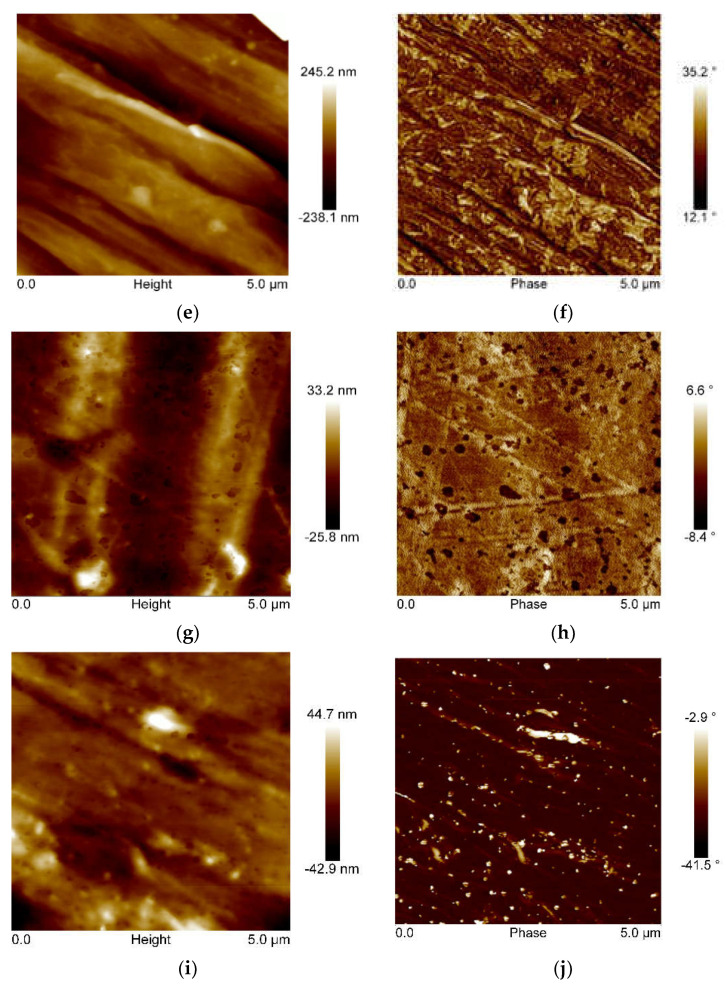
AFM images showing topographies for: (**a**) PET-G, (**c**) PET-G/3%S, (**e**) PET-G/3%B, (**g**) PET-G/3% L, and (**i**) PET-G/1,5%L/1.5%B; and phase images for: (**b**) PET-G, (**d**) PET-G/3%S, (**f**) PET-G/3%B, (**h**) PET-G/3%L, and (**j**) PET-G/1.5%L/1.5%B.

Observing the obtained test results presented in Figure 6, it was found that the addition of modified S, B, and L fillers and the L/B hybrid system significantly changed the surface of the composite. The observed effect was also confirmed by the roughness of the tested materials determined during the analysis, particularly the Rα parameter (mean standard deviation of the profile from the baseline), which was increased by 1.45–9.08 nm in relation to the unmodified matrix (Table 5). For the PET-G/3%B composite, the best roughness was obtained as compared to the unmodified PET-G polymer matrix.

The presented phase images also showed changes in the structure of the composite (Figure 6). For the surface of unmodified PET-G, the image was smoothened and it was impossible to distinguish two distinct phases. On the other hand, observing the results of the AFM analysis performed for composites, two phases were distinguished: polymer-filler. In the obtained phase image for PET-G/3%S, dark brown spots from the dispersed additive on the surface of the sample were noticed, as well as light brown areas indicating the presence of a filler surrounded by the polymer (Figure 6d). Dark brown clusters indicate the formation of small agglomerates of irregular shapes with an average size of 159.69 nm. For the PET-G/3%B composite, a layered topography was obtained, which is characterized by a lamellar arrangement of the filler (Figure 6e). In the phase image, evenly distributed bentonite plates (bright area) were observed, the size of which did not exceed 903.85 nm (Figure 6f). Figure 6h shows well-dispersed filler L in the form of dark areas, which was characterized by a fibrous shape and particle size in the range from 22.02 nm to 341.42 nm. The introduction of the hybrid L/B system resulted in obtaining a surface on which the layered arrangement of the topography is visible (Figure 6i). On the other hand, proper dispersion of the L/B filler in the PET-G polymer matrix was observed. The analysis of the surface size of the tested composites confirmed the nanometric size of the introduced nanoadditives [33,34,35]. On this basis, an appropriate dispersion of the fillers used in the polymer matrix was found, which proves that the homogenization process was correctly carried out.

**Figure 7:**
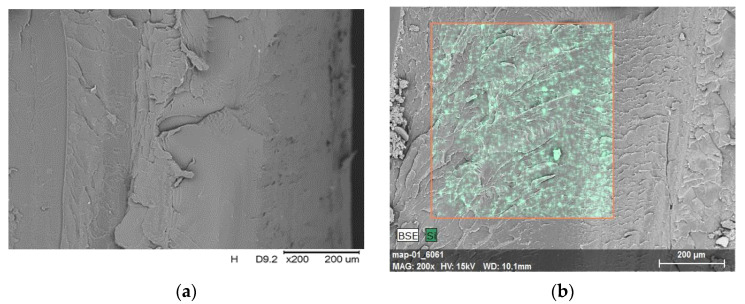

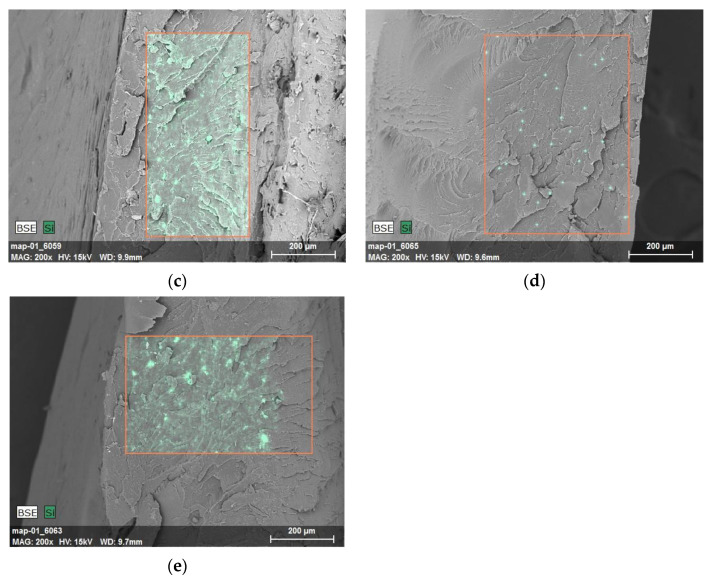
SEM micrographs with an EDS attachment of PET-G polymer and composites based on PET-G: (**a**) PET-G, (**b**) PET-G/3%S, (**c**) PET-G/3%B, (**d**) PET-G/3%L, and (**e**) PET-G/1.5%L/1.5%B. The red contour marks the area subjected to EDS analysis, which was performed in order to observe the degree of filler dispersion and the distribution of the silicon element.

The morphology of brittle fractures of the obtained composites was analyzed with the use of scanning electron microscopy (SEM) with an energy-dispersive spectroscopy, EDS, microanalysis device. The breakthroughs were obtained after cooling the samples in dry ice and their impact fracture, and the results of these observations are presented in Figure 7a–e.

The presence of a few tabs on the brittle fracture surface of the unfilled polymer matrix was observed (Figure 7a). The introduced modified fillers did not affect the surface characteristics; only in the case of PET-G/3%B were more visible furrows obtained (Figure 7c). The distribution of the Si element in the marked area was determined, and the results are summarized in Figure 4. Analyzing the obtained SEM/EDS imaging results for PET-G/3%S (Figure 7b) and PET-G/3%B (Figure 7c), the correct distribution of the filler in the polymer matrix was confirmed. In the case of a composite containing a hybrid system of fillers (1.5%L/1.5%B), there were a few noticeable clusters of these additives (Figure 7e). On the other hand, for PET-G/3%L (Figure 7d), a small amount of the Si element was revealed on the surface of the composite in the area selected for testing

**Figure 8:**
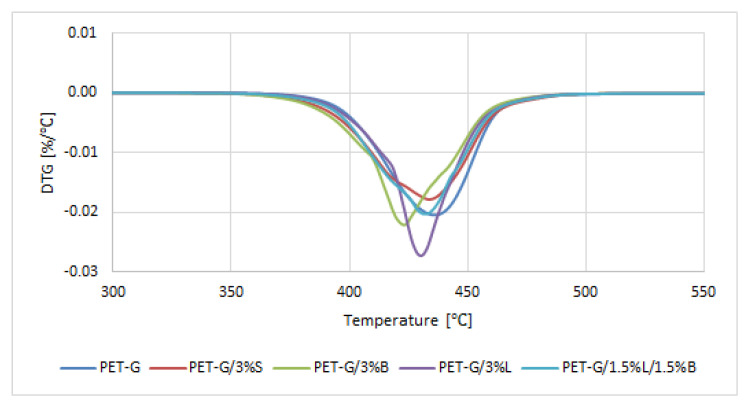
Results of DTG analysis (mass change derivative curve) of PET-G polymer and composites based on PET-G.

The results of testing the thermal stability properties of the composites are summarized in Table 6 and Figure 8. The temperature of 5% weight loss (T_5%_) was determined from the TGA curve, which can be taken as the beginning of the degradation process. The maximum temperature of the degradation steps (T_1_) was determined from the mass change derivative curve.

The obtained composites were characterized by a single-stage thermal decomposition, and the most intense peak was obtained for the PET-G/3%L composite (Figure 8). Polymer composites degraded in the temperature range of 360–490 °C. The unmodified polymer had the best thermal stability (Table 6). It was observed that PET-G generated a large amount of carbon residue at the temperature of 600 °C, which is also confirmed by the literature [36,37]. The introduced modified fillers slightly decreased the thermal stability of the polymer (Table 6).

**Figure 9:**
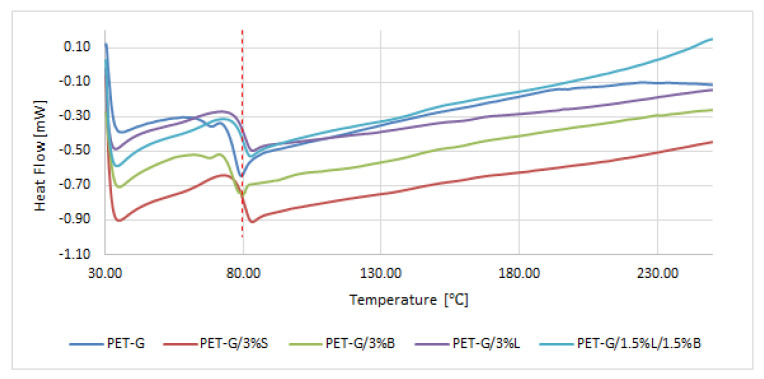
Results of the differential scanning calorimetry (DSC) analysis of PET-G and PET-G composites.

In the next stage of the work, DSC analysis of glycol-modified poly (ethylene terephthalate) and composites on its matrix was carried out. It was observed that the tested materials were characterized by one inflection (Figure 9). Inflection occurred at a temperature of about 80 °C, which corresponds to the glass transition temperature (T_g_) of PET-G [38,39]. The introduction of modified S, B, and L fillers and the hybrid L/B system did not cause significant changes in the thermal history of PET-G, because the T_g_ was obtained for the composites in the range of 78.89–79.91 °C, while for the unfilled polymer matrix, it was obtained at exactly 79.84 °C.

**Figure 10:**
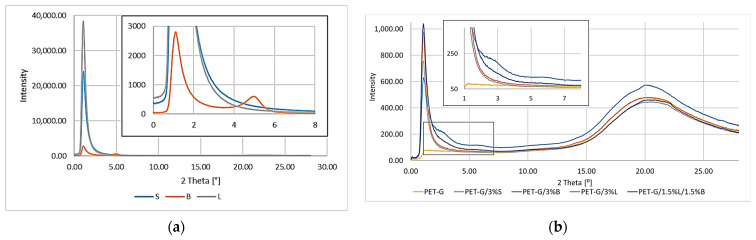
WAXS patterns: (**a**) fillers; (**b**) PET-G and composites with the addition of modified S, B, and L fillers.

The morphology and molecular orientation of the composites and fillers were characterized by WAXS analysis, and the plots of radiation intensity as a function of scattering angle are shown in Figure 10a,b.

The scattering angle for the tested materials was in the range from 0° to 28°. Due to the capabilities of the apparatus, for fillers in this range, it was only possible to observe the peak for the modified bentonite B (Figure 10a).

The distance between successive planes of the filler (d_hkl_) was calculated from the Bragg formula:

(1) $$d_{\mathrm{hkl}}=\frac{n\lambda }{2sin\theta }$$

where n is the degree of diffraction (*n* = 1, 2...), λ is the wavelength of radiation used, and 2θ is the angle at which the diffractive peak occurs, as read from the WAXS graph.

The particle size in the Scherrer formula was also determined:

(2) $$D_{\mathrm{hkl}}=\frac{K\lambda }{bcos\theta }$$

where D_hkl_ is the reflex width dependent on the size of crystallites, K is Scherrer’s constant, K = 1, λ is the wavelength of radiation used, and b is the half-width of the diffraction peak for the plane (_hkl_).

Analyzing the results obtained for the fillers (Figure 10a), we can see a peak for B at 4.98°, which can be attributed to the diffraction reflection from the (001) bentonite sheets [40]. Unfortunately, it was not possible to determine the characteristic peaks for the remaining modified S and L fillers due to the applied assay conditions and technical capabilities of the apparatus. The distances between successive packets of filler plates and the size of their particles were calculated; for B d_khl_, it was 18.20 Å, while for D_khl_, it was 110.8 Å. 

PET-G and the obtained composites were characterized by a broad peak at the value of 2*θ* of about 20°, which was assigned to PET-G. The introduced fillers were properly dispersed in the matrix as evidenced by the lack of additional peaks, the exception being the PET-G/3%B composite (Figure 10b). The band for filler B was disclosed at 4.98° (Figure 10a), while when analyzing the results for the PET-G matrix composite containing the modified bentonite, no band was observed at this value. For PET-G/3%B, only an additional peak could be distinguished at the value of 2*θ* equal to 2.81°, which proved that the bentonite plates were spaced apart. In order to confirm the dispersion of the filler, the distances between successive packets of B plates and the size of their particles were calculated; for B d_khl_, it was 32.24 Å, while for D_khl_, it was 62.55 Å. Comparing the obtained results of d_khl_ and D_khl_ with those calculated for the filler, an increase in the distance between the bundles of bentonite plates by 14.04 Å and a decrease in particle size by 48.25 Å, which is related to the homogenization in a twin-screw extruder, was observed.

**Figure 11:**
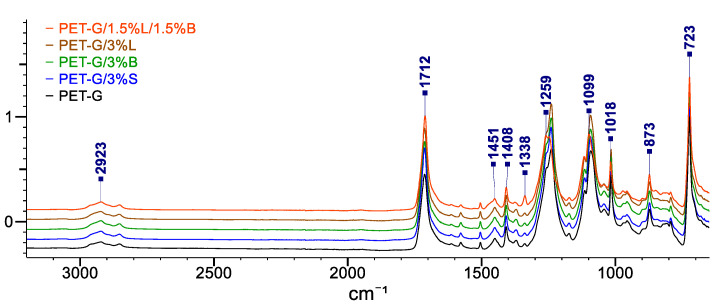
FT-IR spectra recorded for the composition.

The obtained FT-IR spectra for PET-G and its composites are presented in Figure 11.

The PET-G spectrum shows absorbance peaks at 2923 cm^− 1^, which can be attributed to CH_2_ stretching vibrations, and at 1712 cm^− 1^ and 1259 cm^− 1^, which correspond to =C=O and =C(=O)O ester groups, respectively. In contrast, peaks at wavelengths of 1451 cm^−1^, 1408 cm^−1^, and 1338 cm^−1^ can be attributed to methylene deformation -CH_2_ vibrations, and the CO bond appears as a peak at 1099 cm^−1^. Moreover, the C–H stretching peak of the cyclohexene ring was found at 1018 cm^−1^ and 873 cm^−1^. The deformation outside the C–H plane of two carbonyl substituents in the aromatic ring appeared as a strong band at a wavelength of 723 cm^−1^ [23,41,42]. All signals characteristic for PET-G were also visible in the obtained spectra of composites on its matrix. No new bands were observed in the spectrum that could come from the chemical bonds of the added fillers.

## 4. Conclusions

Currently, on the basis of literature data, we observe a dynamic development of rapid prototyping techniques, particularly those based on the methods of joining extruded molten filaments. Therefore, the need for new polymer materials with better-performance properties is clearly growing. Unfortunately, a large number of publications mainly present the properties of elements made of unmodified basic materials. The use of hybrid polymer materials can unleash the efficiency of additive manufacturing and can address basic process characteristics such as printability (flowability and viscosity) of the material. Therefore, this work supplements the information on innovative polymer composites dedicated to 3D printing technology that meet the high requirements for this type of material. For this purpose, research was carried out on the development of polymer composites with a PET-G matrix with the addition of modified nanofillers dedicated to 3D printing in MEM technology. Several fillers, known and described in the literature, were selected and dispersed in the polymer matrix, and then the fibers were obtained on a specially designed and developed technological line. The influence of fillers, including silica modified with alumina, bentonite modified with quaternary ammonium salt, and a hybrid lignin/silicon dioxide filler system, on the properties of the obtained composites was investigated. It was found that the addition of modified fillers to the PET-G matrix increased the flowability (MFR) and thus decreased the viscosity of the material. The highest melt flow rate result was obtained for the PET-G/3%S composite, where the change compared to the unfilled polymer was 45.63%. On the other hand, the lowest viscosity composite was PET-G/3%B, which was matched by the PET-G/3%S composite at higher shear rates. An increase in the Rockwell hardness of the obtained composites was observed, both for injection-molded and 3D-printed samples, which is directly related to the decrease in the impact toughness of these materials. It was also observed that the material stiffness increased with the addition of modified nanofillers, as evidenced by the increase in Young’s modulus for the sample, regardless of the production technique, except for the samples PET-G/3%S (1327.17 MPa) and PET-G/3%L (1381.47 MPa) obtained by 3D printing. Observations of the microstructure of composites using the SEM/EDS method, as well as AFM, confirmed the nanometric size of the fillers and their uniform distribution in the polymer matrix, which was also observed on the basis of the WAXS analysis results. The roughness results indicated that the introduced fillers affected the structure of the samples, as an increase in the Ra parameter was observed in the range from 48.01% (PET-G/1.5%L/1.5%B) to 300.66% (PET-G/3%B) compared to unfilled polymer. The TGA results showed that the addition of fillers reduced the thermal stability of the composites, and the material with the lowest thermal stability turned out to be PET-G/3%B. The DSC study showed that PET-G was characterized by phase changes typical for the material, and the added additives did not change the thermal history of the composites. The spectrum obtained for the polymer (FT-IR) contained all the characteristic functional groups of the material, and the introduced fillers did not affect the distribution of the bands obtained.

## Figures and Tables

**Table 1:** Compositional data of the composites.

Composition	PET-G Content (wt.%)	S Content (wt.%)	L Content (wt.%)	B Content (wt.%)	E926 Content (wt.%)
PET-G	100	-	-	-	-
PET-G/3%S	96	3	-	-	1
PET-G/3%B	96	-	-	3	1
PET-G/3%L	96	-	3	-	1
PET-G/1.5%L/1.5%B	96	-	1.5	1.5	1

**Table 2:** Selected printing and injection parameters.

Printing Parameters		Injection Parameters	Paddles	Bars
Nozzle diameter, mm	0.4	Mold temperature, °C	70	70
Layer height, mm	0.2	Injection temperature, °C	220	220
Infill percentage, %	100	Injection pressure, bar	600	750
Infill pattern, °	±45	Post pressure, bar	550	700
Extrusion temperature, °C	220	Plasticizing time, s	120	120
Bed temperature, °C	80	Injection time, s	5	5
Printing speeds, mm/s	70	Post time, s	3	3

**Table 3:** Summary of the obtained MFR results.

Composition	PET-G	PET-G/3%S	PET-G/3%B	PET-G/3%L	PET-G/1.5%L/1.5%B
MFR (g/10 min)	3.53 ± 0.01	5.17 ± 0.06	4.28 ± 0.02	4.93 ± 0.03	4.95 ± 0.09

± standard deviation.

**Table 4:** Results of mechanical tests of PET-G and composites based on PET-G.

Composition	Young’s Modulus [MPa]	Stress at Break [MPa]	Strain at Break [%]	Young’s Modulus [MPa]	Stress at Break [MPa]	Strain at Break [%]
	3D printing			Injection		
PET-G	**1361.62** *±26.12*	**22.56** *±1.56*	**9.04** *±0.12*	**1545.65** *±16.16*	**29.53** *±0.85*	**19.94** *±0.21*
PET-G/3%S	**1327.17** *±24.17*	**19.84** *±5.34*	**9.58** *±2.48*	**1563.72** *±35.72*	**28.70** *±0.78*	**21.73** *±5.96*
PET-G/3%B	**1415.44** *±73.86*	**35.09** *±8.46*	**7.68** *±1.84*	**1643.69** *±9.48*	**28.32** *±0.66*	**19.99** *±3.02*
PET-G/3%L	**1317.01** *±47.11*	**33.98** *±0.82*	**7.14** *±0.64*	**1578.13** *±1.42*	**28.94** *±0.27*	**20.55** *±0.60*
PET-G/ 1.5%L/1.5%B	**1381.47** *±34.93*	**32.36** *±2.26*	**7.21** *±1.09*	**1620.81** *±31.32*	**28.28** *±0.21*	**27.72** *±7.92*

**Table 5:** Summary of the obtained MFR results.

Composition	PET-G	PET-G/3%S	PET-G/3%B	PET-G/3%L	PET-G/1.5%L/1.5%B
Rα [nm]	3.02	6.19	12.1	5.51	4.47

**Table 6:** The results of research on the properties of thermostability of composites.

Composition	T_2%_ [℃]	T_5%_ [℃]	T_1_ [℃]	ΔV_1_ [%/℃]	R_600_ [%]
PET-G	385.99	400.31	435.98	0.02	8.65
PET-G/3%S	381.99	394.09	433.78	0.02	11.43
PET-G/3%B	378.09	390.68	423.20	0.02	9.83
PET-G/3%L	389.89	400.27	430.21	0.03	9.80
PET-G/1.5%L/1.5%B	385.05	397.47	432.11	0.02	11.38

## Data Availability

The data presented in this study are available on request from the corresponding author.
